# Assessment of Maternal Health Services Quality at Juba Teaching Hospital, South Sudan

**DOI:** 10.24248/eahrj.v4i1.620

**Published:** 2020-06-26

**Authors:** William Ezbon Apary, Dinah Amongin

**Affiliations:** a Clarke International University, Kampala, Uganda

## Abstract

**Background:**

Client satisfaction is an important outcome of healthcare services and is regarded as an indicator for quality of care. Not much research has been conducted to investigate satisfaction with maternal health care in Sub-Saharan Africa and especially no or little in South Sudan. This study was carried out to assess quality of maternal health services (MHS) at Juba Teaching Hospital, South Sudan.

**Methods:**

This cross-sectional research study was done among women of reproductive age at a time of taking their infants for routine immunization services in June to July 2015. A structured questionnaire was used to gather information by interviewers. The data were analysed using SPSS Statistics v20. The frequency tables were for describing data and chi-square test and logistic regression were used to determine whether there was statistical association of sociodemographic factors with satisfaction with MHS.

**Results:**

Of 207 women, 193 (93.0%) were satisfied with the services. There was statistically significant association of family monthly income with antenatal careand delivery care satisfaction [OR at 95%CI = 8.30 (2.04 – 33.79), p-value=0.003 and OR at 95%CI = 0.12 (0.03 – 0.56), p-value = 0.007 respectively]. Furthermore, there was a significant association of education attainment with place of delivery [OR at 95%CI =3.06 (1.40 – 6.71), p-value = 0.005]

**Conclusion:**

Women were satisfied with maternal healthcare services. Level of education and monthly family earnings were associated with maternity care. Hence, there should be emphasis on the education of women and their earnings.

## BACKGROUND

Maternal health service (MHS) is a set of services related to maternity health care. These health services cause a serious concern worldwide. Among others, MHS incorporates antenatal care, delivery care and postnatal health service.

Global Statistics stood at 289,000 maternal deaths in 2013.^1^ The developing nations accounted for 289,000 (99.0%) with region of sub-Saharan Africa (SSA) alone accounting for 62.0% (179,000) of global deaths followed by Southern Asia at 69,000 (24.0%).^1^ For South Sudan, there was a substantial estimation of about 3,000 maternal deaths as of the year 2013.^1^

While the proportion of pregnant women in developing countries with attendance of four antenatal clinic (ANC) visits is estimated at about 52.0%, the low-income countries also stood at around 38.0% of pregnant women who attended four times or more ANC visits.^2^ For South Sudan around 46.7% of pregnant women attended one ANC visit.^3^

It has been globally reported that 66.0% of mothers attended delivery care at health facilities.^4^ In developing countries, there were 53.0% of pregnant mothers who gave birth at health facilities.^5^ This proportion for the SSA^6^ and South Sudan estimated at 40.0% and 14.0% respectively.^6^

Importantly, It has been shown that there has been no or little literature on the proportion of postnatal care at global, regional and national levels. Nevertheless, this was estimated at about 30.3% for developing countries.^5^

South Sudan has one of the highest maternal mortality rates in the world and this necessitated the greater attention to maternal and child health services in the country. This attention is apparently indicated in its strategic development plan: “*To increase the utilization and quality of health services with emphasis on maternal … health.*”^3^ South Sudan maternal deaths are estimated at 2054 per 100,000 live births^7^. South Sudan maternal deaths estimated at 3000. The maternal deaths have substantially increased by 46.1% between 2013 and 2014.

Ministry of Health Republic of South Sudan envisions “*a healthy and productive population, fully exercising its human potentials*”, with the mission of providing quality healthcare to all the people of the nation, especially most vulnerable women and children.^3^

Juba Teaching Hospital (JTH) is one of the government institutes implementing quality healthcare in South Sudan.^3^. The hospital serves Central Equatoria State and acts as highest referral hospital for entire South Sudan, which has about 8.26 million people.^3^ The services include, but not limited to ANC, delivery and postnatal care (PNC).^3^ Despite Ministry of Health Republic of South Sudan and its health partner's interventions on quality of mother and child health (MCH) services around 46.7% of pregnant women attended ANC visit.

Despite Ministry of Health Republic of South Sudan and its health partner's interventions on quality of mother and child health (MCH) services around 46.7% of pregnant women attended ANC visit. Skilled health professionals attend only 14.7% of deliveries and institutional deliveries account for about 12.3% of births.^3^ This could be due to quality issues of MHS and in particular the ANC, delivery and PNC services. Unfortunately, the level of quality of MHS and postnatal care in South Sudan are unknown. Consequently, there is paucity of information regarding the proportion of MHS utilization among mothers accessing Juba Teaching Hospital (ANC, delivery and PNC), the level of mothers' satisfaction towards the quality of MHS among mothers attending at Juba Teaching Hospital and mothers' demographic factors influencing their satisfaction with quality of MHS. These limitations of quality in maternal health care could explain the observed increase in mortality in South Sudan.

This study assessed the quality of MHS quality among women accessing the Juba Teaching Hospital from June to July 2015. Findings obtained would fill this knowledge gap and would be useful for objective and evidence-based decision making by the Ministry of Health, Republic of South Sudan and its health development partners. Moreover, good practices and lesson learned would be nationally established and used to improve health delivery services at large as well as to enhance the health of mothers in particular.

### Objectives

The main objective was to assess the quality of MHS at Juba Teaching Hospital in South Sudan. The specific objectives were to establish the level of mothers' satisfaction towards the quality of MHS and to determine mothers' socio-demographic factors influencing their satisfaction with quality of MHS.

## METHODS

### Research Design and Rationale

The study design was cross-sectional health facility based to assess the quality of MHS in 2015. The cross-sectional study design produces valid research outcomes particularly when the research has been about quality of care.^5, 8, 9^

### Study Site

This study was carried out in JTH in 2015. The hospital is a government health facility which is situated in Juba city of Central Equatoria State. Juba city also is served as national capital city of South Sudan. The study was conducted in Juba with anticipation that the findings obtained would be incorporated into health policy and translated into useful and implementable health strategies for improvement of MHS. In addition, the choice of JTH was also due to its accessibility. Sudan Government established this teaching hospital in order to serve medical students of the health college of the University of Juba.^26^

South Sudan has since adopted this health facility as its main national referral hospital after its secession from Sudan in a referendum that resulted in its declaration of independence as of July 2011. JTH now serves not only as medical school teaching hospital, but also as a highest referral hospital for the entire country of South Sudan.^26^ The hospital offers health service that range from primary to tertiary health care. These services include, but not limited to antenatal care, delivery care, and post-natal services. Furthermore, it provides expanded program for immunisation, pharmaceutical services, health promotion as well as intensive care, accident and emergency services, voluntary counselling and testing of HIV/AIDs, caesarian section services among others^27, 28^

### Study Population

The study population were women aged between 15 and 49 years of age who were accessing MCH services at JTH during in 2015.

### Eligibility Criteria

**Inclusion Criteria:** Consenting women who were between 18 and 49 years of age with infants; were accessing child health-care, the routine immunisation services at JTH; and were with parity of two or more were eligible for this study.

**Exclusion Criteria:** Women who declined to consent were excluded from participation in this study.

### Sample Size

While the outcome of this research appeared to be categorical data, the following formulae were appropriate to be applied:

(1) $${\text{n}}_{0}=\left[{\text{Z}}^{2}\text{p}\left(1-\text{p}\right)\right]/{\text{d}}^{2}$$

where

n_0_ ≡ initial estimated sample size

Z ≡ level of statistical confidence interval at 95% that has standard value of 1.96

P ≡ proportion of satisfaction with MHS was set to be 50% or 0.5. This was because, it was unknown.

d ≡ degree of accuracy that was required, that is, margin of error =5% or 0.05

Thus, n_0_ = [(1.96)^2^ * 0.5 (1 – 0.5) ] / (0.05)^2^ = 384

Initially, the study participants were 384 according to the estimation of the above formula for infinite population.

However, the population of mothers who were accessing the routine immunisation services at Juba Teaching Hospital were known or their sample frame 403 women. Hence, the following finite population correction factor for proportion was applied:

(2) $$\text{n}={\text{n}}_{0}/\left[1+\left({\text{n}}_{0}/\text{N}\right)\right]$$

where

n ≡ estimated sample size

n_0_≡ initial estimated sample size, that was 384 women

N≡ Sample frame, that was 403 women. These were estimated number of women who registered their children for routine immunization at Juba Teaching Hospital. As a result of the preceding formulae, the representative sample size was 196.68 women. This figure was round into 197 women. This was because the women are discrete data, not continuous data.

With the margin of error was 5%, the sample size was brought about to 207.

### Sampling Technique

A simple random sampling procedure was used in selecting the study participants attending routine immunisation service at JTH in South Sudan. This procedure was applied because the participants of this research study were homogeneous. In the procedure, the women were listed by numbering them. Simple random tables were used so as to eliminate the bias in recruiting the study participants. The research assistants interviewed the selected study participants using structured questionnaire. As soon as the interview was finished with one woman, the next was interviewed. The language used in the interviews was Arabic.

### Study Unit

The Study unit was an individual woman who has accessed the routine immunisation at JTH in South Sudan.

### Study Variables

The independent variables were age, religion, education level, occupation, employment status, family income per month in South Sudanese Pound (SSP), marital status, type of marriage and parity. The dependent variable was level of mothers' satisfaction with MHS (ANC, delivery care and PNC).

### Data Source

The source of data was primary data interviewing the women at the routine immunisation at JTH.

### Data Collection Techniques and Instrument

The face-to-face interview was conducted in collecting data by research assistants that were recruited based on their competencies in administering questionnaire, conducting interview as well as previous experience in this context. At routine immunization service delivery at JTH, eligible women were interviewed. The purpose, objectives and method of the study were explained to each woman. They were assured that the research did not carry any physical harm. Furthermore, the high degree of confidentiality and privacy were also guaranteed and particularly anonymity of their responses in the entire research process. Afterwards, informed consent was received by signing on the consent form. This interview procedure was conducted repeatedly until the process of data collection was completed.

The tool that was used in collecting data was structured questionnaire which was drawn from three previously used questionnaires. These were adapted to this research study. Some questions were derived from previous relevant studies.^10^ Other questions were drawn from research study about client satisfaction with heathcare.^11, 12^

The data collection tool was organized into two sections: Section I: Demographic factors; Section II: MHS utilization and satisfaction (antenatal care, delivery care and postnatal service). The women of reproductive age were requested to rate the level of their satisfaction with the services using five (5) scale ranging from very poor to excellent.

### Data Management and Analysis Techniques

Using EpiData version 3.1, the software questionnaire was designed, prepared and checked (legal range, jump, must enter value label). This designing process was carried out after the data had been cleaned up for omission and errors during the data collection process. After wards, the data were entered into the EpiData (CDC, USA) so as to form database (rec) that was exported into statistical package for social science (SPSS statistics, IBM, USA) data base (spv) for analysis phase.

Since the outcome of this study was categorical variables, cross tabulation and logistic regression were conducted using SPSS statistic version 20. The process was carried out in order to check up for chi-square test, Fisher exact values, Spearman correlation, odd ratios, confidence interval at 95% level, P-values; and to determine whether there was an association between sociodemographic factors with maternal healthcare satisfaction. This stage was also conducted after some incompatible data were transformed into dichotomous variables. The data were validated and reduced for logistic regression analyses. Furthermore, frequencies and percentages were carried out. For the numeric variables, mean and range were summarised.

### Quality Control Techniques

The quality control procedures consisted of a preliminary visit, training of research assistants and data management.

Both preliminary visit to the hospital and the Ministry of Health were conducted so as to get acquainted with ethical procedures of conducting the research study.

Training of research assistant on the study protocol was carried out for five days, with emphasis on the use of random tables and conducting interview.

Pre-test of the questionnaire were carried out for 5% of the sample size at the hospital. It was found that the questionnaire was well understood therefore remained unchanged. The pre-test findings were included in the analysis.

Quality of data was ensured by checking for quantitative data completeness, clearing, entering into Epidata; data validating, coding, and transforming until they were presentable for the analysis.

### Ethical Consideration

The ethical consideration is significant particularly the research study involving human participants. This consideration was constituted of several stages.

To start with, the official letter was obtained from the university – International Health Science for conducting this research after the research proposal was reached.

Afterwards, the letter and seven (7) hard copies of the research proposal were submitted to the Ethical Committee at the Ministry of Health Republic of South Sudan for close review.

Subsequently; while the Ministry of Health acknowledged the importance of the study proposal to fill the gaps in knowledge to improve the health care provision for mothers of reproductive age, the proposal was authorized to be conducted (Ethical letter reference number MOH-RSS/15/07/014).

At Juba Teaching Hospital; the purpose, objectives and significance of the study were clarified to each study participant. Furthermore; the risk, privacy and confidentiality of conducting the research were surely explained to each woman of childbearing age. Then, the opportunity was given for the questions. Accordingly, the feedback was provided.

Finally, the face-to-face interview was administered in a conducive environment after the informed consent was obtained by signature or thumb print.

## RESULTS

### Sociodemographic Factors of the Mothers

The Table 4.1 shows descriptive analysis of the mothers' demographic characteristics. A total of 207 women were interviewed with the response rate of 100%. The mean age of the respondents was 27±4.9, the yougest was 19 years old and the oldest was 42 years old. The proportion of the participants within the range of 18 to 35 years of age was the highest (94.2%).

**TABLE 4.1:** Sociodemographic Factors of the Mothers

Sociodemographic factors	n=207	Percent
Age*		
18-35years	195	94.2
35-49years	12	5.8
**Religion**		
Christian	187	90.3
Islam	18	8.7
Others	2	1.0
**Level of education**		
No formal education	49	23.7
Primary education	78	37.7
Secondary education	63	30.4
Tertiary education	17	8.2
**Occupation**		
Housewife	184	88.9
Others	23	11.1
**Employment status**		
Employed	71	34.3
Unemployed	110	53.1
Self-employed	26	12.6
Monthly income*		
<US$300	94	45.4
US$300-600	76	36.7
US$600-900	16	7.7
US$900-1200	16	7.7
>US$1200	5	2.4
**Marital status**		
Married	202	97.6
Separated/widow	3	1.4
Others	2	1.0
**Type of marriage**		
Monogamy	134	64.7
Polygamy	73	35.3
**Parity**		
Multipara	177	85.5
Grand multipara	30	14.5

* upper included in next category

With regard to level of education, 158 of the mothers (76.3%) had gone through primary education with only 8.2 % having attained higher education. Unemployment accounted for more than half (53.1%) of the women while low proportion of them (12.5%) reported to have had a self-employment. The mean monthly income of the family was US$407.81; the lowest family income per month was US$15.6 and the highest was US$2821.32 with very wide standard deviation (US$328.26). There were 202 (97.4%) married women and approximately two-third (64.7%) of them indicated to have had a monogamous union. Most of them (85.5%) were multipara or parity of 2 to 4 births. The mean parity of the mothers as 3.15±1.52. The lowest figure was 2 and the highest was 9 births.

### Level of Mothers' Satisfaction with MHS

The results displayed in Table 4.2 show counts and proportions of overall satisfaction, dissatisfied/satisfied with antenatal, delivery and postnatal care. Of the 142 mothers, 132 (92.9%) were satisfied with ANC while 10 (7.0%) were dissatisfied. Regarding the satisfactory level with ANC, good was the highest score, 81 (57.0%).

**TABLE 4.2.** Level of Mothers' Satisfaction With Maternal Health Services

Variables	n=207	Percent
ANC overall satisfaction^*^		
Dissatisfied	10	7
Satisfied	132	93
Delivery overall satisfaction^**^		
Dissatisfied	10	6.8
Satisfied	136	93.2
PNC overall satisfaction^***^		
Dissatisfied	16	8
Satisfied	184	92
Level of ANC satisfactory^*^		
Very poor	6	4.2
Poor	4	2.8
Good	81	57
Very good	33	23.2
Excellent	18	12.7
Level of delivery satisfactory^**^		
Very poor	4	2.7
Poor	6	4.1
Good	84	57.5
Very good	37	25.3
Excellent	15	10.3
Level of PNC satisfactory^***^		
Very poor	6	3
Poor	10	5
Good	119	59.5
Very good	40	20
Excellent	25	12.5
**Maternal care satisfaction**		
Satisfied	193	93
Dissatisfied	14	7

Variation within n (

* n = 142,

** n = 146,

*** n = 200) was due to systemic exclusion using questionnaire. Satisfied = Excellent, very good and good. Dissatisfied = poor and very poor

Out of the 146 women, only 10 (6.8%) were dissatisfied with the delivery care whereas 136 (93.1%) were satisfied. Of those women, good also emerged to be the highest degree of satisfaction, 84 (57.5%) in terms of delivery care.

Of the 200 mothers who received postnatal care services, 184 (92.0%) were satisfied while 16 (8.0%) were dissatisfied.

### The Influence of Demographic Factors on Satisfaction With MHS

The Tables 4.3 and 4.4 illustrate bivariate analysis of mothers' demographic factors that determine the satisfaction with antenatal care, delivery care and postnatal service, respectively. Cross tabulations were used whereby Pearson Chi-Square, Fisher's Exact Test and Spearman correlation were conducted.

**TABLE 4.3:** Results of Relation Between the Demographic Factors and Satisfaction With MSH

Variables	Delivery Overall Satisfaction		Total: n (%)	P Value
	dissatisfied: n (%)	satisfied: n (%)		
**Age group**				.430
18-35years	10 (100)	128 (94.1)	138 (94.5)	
35-49years	0 (0.0)	8 (5.9)	8 (5.5)	
**Religion**				.430
Christian	8 (80.0)	124 (91.2)	132 (90.4)	
Islam	2 (20.0)	11 (8.1)	13 (8.9)	
Others	0 (0.0)	1 (0.7)	1 (0.7))	
**Level of education**				.317
No formal education	0 (0.0)	26 (19.1)	26 (17.8)	
Primary education	4 (40.0)	54 (39.7)	58 (39.7)	
Secondary education	4 (40.0)	45 (33.1)	49 (33.6)	
Tertiary education	2 (20.0)	11 (8.1)	13 (8.9)	
**Occupation**				.832
Housewife	9 (90.0)	125 (91.9)	134 (91.8)	
Others	1 (10.0)	11 (8.1)	12 (8.2)	
**Employment status**				.444
Employed	2 (20.0)	50 (36.8)	52 (35.6)	
Unemployed	6 (60.0)	72 (52.9)	78 (53.4)	
Self-employed	2 (20.0)	14 (10.3)	16(10.9)	
**Monthly family income**				.004*
<US$300	2 (20.0)	67 (49.3)	69 (47.3))	
US$300-600	3 (30.0)	52 (38.3)	55 (37.7)	
US$600-900	4 (40.0)	8 (5.9)	12 (8.2)	
US$900-1200	1 (10.0)	8 (5.9)	9 (6.2)	
>US$1200	0 (0.0)	1 (0.7)	1 (0.7)	
**Marital status**				.860
married	10 (100)	132 (97.1)	142 (97.3)	
Separated/widow	0 (0.0)	3 (2.2)	3 (2.1)	
others	0 (0.0)	1 (0.7)	1 (0.7))	
**Type of marriage**				.111
Monogamy	9 (99.0)	89 (65.4)	98 (67.1)	
Polygamy	1 (10.0)	47 (34.6)	48 (32.9)	
**Parity**				.343
multipara	8 (80.0)	122 (89.7)	130 (89.1)	
grand multipara	2 (20.0)	14 (10.3)	16 (10.9)	

* P value < 0.05

**TABLE 4.4:** Results of Relation Between the Demograhic Factors and Satisfaction With PNC

Variables	PNC Overall Satisfaction		Total: n (%)	P Value
	dissatisfied: n (%)	satisfied: n (%)		
**Age group**				.292
18-35years	16 (100)	172 (93.5)	188 (94.0)	
35-49years	0 (0.0)	12 (6.5)	12 (6.0)	
**Religion**				.862
Christian	15 (93.8)	166 (90.2)	181 (90.5)	
Islam	1 (6.8)	16 (8.7)	17 (8.5)	
Others	0 (0.0)	2 (1.1)	2 (1.0)	
**Level of education**				.495
No formal education	2 (12.5)	44 (23.9)	46 (23.0)	
Primary education	5 (31.3)	71 (38.6)	76 (38.0)	
Secondary education	7 (43.8)	55 (29.9)	62 (31.0)	
Tertiary education	2 (12.5)	14 (7.6)	16 (8.0)	
**Occupation**				.078
Housewife	12 (75.0)	165 (89.7)	177 (88.5)	
Others	4 (25.0)	19 (10.3)	23 (11.5)	
**Employment status**				.354
Employed	8 (50.0)	61 (33.2)	69 (34.5)	
Unemployed	7 (43.8)	98 (53.3)	105 (52.5)	
Self-employed	1 (6.3)	25 (13.6)	26 (13.0)	
**Monthly family income**				.959
<US$300	8 (50.0)	82 (44.6)	90 (45.0)	
US$300-600	6 (37.5)	68 (37.0)	74 (37.0)	
US$600-900	1 (6.3)	15 (8.2)	16 (8.0)	
US$900-1200	1 (6.3)	14(7.6)	15 (7.5)	
>US$1200	0 (0.0)	5 (2.7)	5 (2.5)	
**Marital status**				.245
married	15 (93.8)	180 (97.8)	195 (97.5)	
Separated/widow	1 (6.3)	2 (1.1)	3 (1.5)	
others	0 (0.0)	2 (1.1)	2 (1.0)	
**Type of marriage**				.155
Monogamy	13 (81.3)	117 (63.6)	130 (65.0)	
Polygamy	3 (18.8)	67 (36.4)	70 (35.0)	
**Parity**				.086
multipara	16 (100)	155 (84.2)	171 (85.5)	
grand multipara	0 (0.0)	29 (15.8)	29 (14.5)	

Almost half [66 (46.5%] of the women who attended ANC had a monthly family income of less than US$300. Of those mothers, 63 (47.7%) were satisfied with ANC while 3 (30.0%) were dissatisfied. The significance of the monthly family income with ANC satisfaction was 0.001 (chi-squire test [df=4, n=207]= 18, P=.001). Since the significance value was less that .05, there had been a statistically significant relation between the monthly family earning and satisfaction with the ANC. This association appeared to be statistically inverse (Spearman correlation = -0.19, P=.024), that is, the lower the family income, the higher the satisfaction with the ANC service.

Similarly, family monthly income had significant association with delivery care service satisfaction (Pearson chi-square [df=4, n= 207] = , P<.05). Of the respondents who gave birth at the Juba Teaching Hospital 146 (70.5%) with the income less than US$300, 49.3% were satisfied; among those with earning between US$300 and US$600, 38.3% were satisfied; and of those with more than US$1200 income, only 0.7% were dissatisfied with the delivery care.

Overall, there was no statistically significant association between the level of satisfaction with MHS and age group, religion, level of education and occupation. There was also no significant association between degree of satisfaction with employment status, marital status, type of marriage and number of children ever born alive to the women.

### Logistic Regression Analysis

There are enormous demographic and health system factors that influence satisfaction of women of reproductive age with maternal healthcare services. In bivariate analysis (shown in the Tables 4.5 and 4.6), it was observed that only monthly family income and level of education were the factors with statistically significant association. Multivariate analysis revealed that 80.3% (114) of the women who had earned less than US$600 on monthly basis were satisfied with the ANC service and were the majority. The significance level and odd ratio with 95% confidence interal were P=.003 and 8.30 (2.04 – 33.79), respectively. There was therefore a statistically significant association between the monthly family income with ANC service. This analysis was consistent with the findings in bivariate analysis.

**TABLE 4.5.** Results of Logistic Regression for Sociodomgraphic Factors and ANC Satisfaction

Variables	n	(%)	OR	(95% CI)	P Value
**Age**					
<30*	112	(78.8)	1.00		
>30	30	(21.1)	2.33	(0.40 - 13.42)	.343
**Religion**					
Christian*	129	(90.8%)	1.00		
Non-Christian	13	(9.2)	1.03	(0.11 - 10.01)	.983
**Education**					
None*	36	(25.4)	1.00		
Literate	106	(74.6)	0.71	(0.15 - 3.32)	.666
**Employment**		1.00			
Yes*	66	(46.5)			
No	76	(53.5)	1.24	(0.29 - 5.29)	.775
**Monthly family income**					
<US$500*	114	(80.3)	1.00		
>US$500	28	(19.7)	8.30	(2.04 - 33.79)	.003**
**Type of marriage**					
Monogamy*	93	(65.5)	1.00		
Polygamy	49	(34.5)	1.27	(0.30 - 5.31)	.745
**Parity**					
<4*	125	(88.0)	1.00		
>4	17	(12.0)	0.28	(0.02 - 3.95)	.345

* reference category,

** P value < 0.05

**TABLE 4.6.** Results of Logistic Regression for Demographic Factors and Delivery Satisfaction

Variables	n	(%)	OR	(95% CI)	P Value
**Age**					
<30	118	(80.8)	1.00		
>30	28	(19.2)	1.26	(0.126 - 12.55)	.844
**Religion**					
Christian	132	(90.4)	1.00		
Non-Christian	14	(9.6)	0.40	(0.06 - 2.45)	.325
**Occupation**					
Housewife	134	(91.8)	1.00		
Others	12	(8.2)	0.83	(0.06 - 11.01)	.887
**Employment**					
Yes	68	(46.6)	1.00		
No	78	(53.4)	0.38	(0.07 - 2.09)	.268
**Monthly family income**					
<US$500	119	(81.5)	1.00		
>US$500	27	(18.5)	0.12	(0.03 - 0.56)	.007**
**Type of marriage**					
Monogamy	98	(67.1)	1.00		
Polygamy	48	(32.9)	6.02	(0.67 - 53.65)	.108
**Parity**					
<4	130	(89.0)	1.00		
>4	16	(11.0)	0.33	(0.03 - 3.18)	.338

* reference category,

** P value < 0.05

The multivariate analysis results indicated that 81.5% (n=119) of the mothers with the same monthly income were satisfied with the delivery care service, P=.007 and odd ratio with confidence interval=0.12 (0.03 – 0.56). Hence, there was statistically significant association that conformed with the bivariate results.

## DISCUSSION

### Level of Satisfaction with MHS

Results of this study indicated that the satisfaction of the women of reproductive age with the quality of MHS was at 93.0%. The satisfaction with quality of ANC, and delivery care were at 93.0% while for PNC services it was at 92.0%. It had been noticed that the rates of satisfaction with ANC and delivery care were similar and slightly higher than that of PNC.

The rate of MHS satisfaction of 93.0% found in this study was higher than that of 76.8% reported in an Egyptian study.^13^ Studies conducted in Bangladesh^14^, Pakistan^15^, India^16^ and Napal^17^ reported satisfaction rates of 62.4%, 61.0%, 51.5%, and 47.8% respectively.

Additionally, in this study satisfaction with the respondents with ANC services was 93.0%. This had a difference of 11.9 % higher than the 81.1% reported in a previous study at a University College Hospital in Nigeria^18^. Furthermore, the satisfaction rate with the ANC services was much higher than those from a research studies undertaken in Western Ethiopian (60.4%)^8^ and Egypt (59.8%).^13^

Furthermore, the results of this research study revealed that satisfaction with DC services stood at 93% which is higher to rates reported in previous studies in Ethiopia (80.7%)^19^, in Egypt (68.7%)^13^ and South Africa (51.9%).^20^

Moreover, satisfaction with PNC was at 92.0% in this study which is also much higher than satisfaction rates found in studies undertaken in South Africa (51.9%)^20^ and in India (22.6%).^16^ Satisfaction level with MHS which combine ANC, DC and PNC services was the highest when compared to findings from previous studies conducted in different developing countries. This observation might explain the high proportions of the utilisation of care recorded, thus affirming the notion that the higher the satisfaction with the quality of maternal healthcare services, the higher the utilisation of the care. This conforms to the hypothesised conceptual framework of this study.

### Determinants of Satisfaction with MHS

There are many sociodemographic and health system factors that influence clients' satisfaction with MHS. This research study revealed only one factor that was monthly family income which had a statistically significant association with MHS. The women with the lower monthly income level were more likely to be satisfied with the quality of MHS than those women with high income. This was consistent with the findings of previous study conducted^21^ on quality of MHS in five states of Nigeria. The authors identified that there was statistically significant association of MHS satisfaction with the income.

Furthermore, it was consistent with empirical evidence of recent research study conducted^8^ on satisfaction with focused ANC service and associated factors among pregnant women attending focused ANC at health centres in Jimma town, Jimma zone, South West Ethiopia. The authors had demonstrated that there was a statistically significant association of income with the satisfaction with the care. Similarly, a study on MHS in Uganda reported that the income was significantly associated with ANC services of antenatal.^22^ However, the results of this study were inconsistent with findings from a previous study on perception and satisfaction with quality of ANC services among pregnant women at the University College Hospital, Ibadan, Nigeria. Those authors had reported that there was no significant association of satisfaction with income.^18^ Additionally, assessment of factors influencing patients' satisfaction with peripartum care at Germiston Hospital Maternity Unit in South Africa revealed that there was no statistically significant association with family monthly income.^23^

Although some findings of this research have shown insignificant statistical association of age, religion devotion, occupation, marital status and parity with satisfaction of the mothers towards quality of MHS, there was a statistical association of education attainment and monthly family earnings with MHS satisfaction. This implies that education attainment and level of income influence satisfaction, which in turn enhances the utilization of MHS. Furthermore, this means that these findings also confirm the hypothesized conceptual framework of this study.

### Study Limitations

This study was health facility based study and therefore limited to only those women who were attended at the health facility. A community based study would have been better so as to incorporate those women who did not go to the health facility. This was response study and has only provided a “*snapshop in time*” of quality of MHS at JTH. Recall bias was another limitation inherent in this type of study but it was partly mitigated by good probing techniques of research assistants during the data collection process.

## CONCLUSION AND RECOMMENDATIONS

### Conclusion

The satisfaction with quality of maternity care was high. The satisfaction with ANC, DC and PNC services were also high. It was established that only a monthly family income had a significant association with ANC and DC services.

### Recommendations

The satisfaction with quality of care is dynamic with time, there is therefore a need to continue improving quality of healthcare that meets the expectations of the women of reproductive age. Since this research did not include data from the community and private health facilities, it is recommended to conduct further research to determine the quality of MHS among women at community level and among mothers in public and private health facilities.
